# Neuroprotective Potential of Broccoli Sprout Extract in Scopolamine-Induced Memory-Impaired Mice

**DOI:** 10.3390/foods14173059

**Published:** 2025-08-29

**Authors:** Huijin Jeong, Hyukjoon Choi, Young-Seo Park

**Affiliations:** 1 Department of Food Science and Biotechnology, Gachon University, Seongnam 13120, Republic of Korea; huijin0218@gmail.com; 2 BK bio, Jeju 63359, Republic of Korea; hjchoi@bkbio.com

**Keywords:** broccoli sprout extract, sulforaphane, scopolamine, neuroprotection

## Abstract

Alzheimer’s disease is characterized by progressive cognitive decline associated with oxidative stress, neuroinflammation, and impaired neurotrophic signaling. Sulforaphane, a bioactive compound found in broccoli, has demonstrated neuroprotective effects by activating NRF2 and inhibiting NF-κB. However, the efficacy of whole-food-derived sulforaphane remains unclear. This study evaluated the neuroprotective potential of broccoli sprout extract using a scopolamine-induced mouse model of memory impairment. Mice were orally administered broccoli sprout extract once daily at doses of 100 mg/kg or 200 mg/kg for four weeks prior to behavioral and biochemical assessments. Treatment with broccoli sprout extract significantly improved scopolamine-induced deficits in long-term memory, as determined by the passive avoidance test. The spatial working memory remained unaffected. High doses of broccoli sprout extract restored hippocampal brain-derived neurotrophic factor levels and reduced cortical lipid peroxidation, suggesting antioxidant and neurotrophic benefits. Additionally, the low dose preserved striatal choline acetyltransferase expression and reduced systemic tumor necrosis factor-alpha and hippocampal cyclooxygenase-2 levels, indicating its anti-inflammatory and cholinergic protective effects. No significant changes in acetylcholinesterase activity or glutathione levels were observed. Overall, these results imply that broccoli sprout extract has multi-targeted neuroprotective effects, possibly involving redox and inflammatory regulation. Therefore, it may be a safe dietary strategy to support cognition in neurodegenerative conditions.

## 1. Introduction

Alzheimer’s disease (AD) is a progressive neurodegenerative disorder marked by cognitive decline, synaptic dysfunction, and neuronal loss. Hallmark pathological features include oxidative stress, neuroinflammation, and diminished levels of neurotrophic factors, particularly in the cortex and hippocampus [1]. Current treatments, such as donepezil, provide only limited symptomatic relief by enhancing cholinergic transmission and fail to alter the underlying disease course [2]. Moreover, these agents are often associated with undesirable side effects [3]. These limitations highlight the urgent need for safer, multi-targeted therapeutic strategies to effectively address the complex pathology of AD.

Sulforaphane, a bioactive isothiocyanate found in broccoli and other cruciferous vegetables, has emerged as a promising neuroprotective compound due to its ability to activate the nuclear factor erythroid 2 (and the related factor 2 (NRF2) pathway) [4]. NRF2 activation leads to the transcription of antioxidants and phase II detoxification enzymes, reducing oxidative stress. Additionally, sulforaphane suppresses neuroinflammation by inhibiting NF-κB-mediated pro-inflammatory cytokine production and promoting anti-inflammatory pathways, including PPARγ activation [5]. Preclinical studies have demonstrated sulforaphane’s ability to protect neuronal integrity and function in models of autism spectrum disorder, epilepsy, AD, and Parkinson’s disease [6]. By simultaneously regulating redox balance and immune responses in the central nervous system, sulforaphane shows strong potential as a preventative or therapeutic intervention for various neurological conditions.

Despite the increasing interest in sulforaphane as a neuroprotective agent, most studies have employed its purified form via non-oral routes, which does not accurately reflect its application in dietary or functional food contexts. Moreover, its bioavailability and pharmacokinetics vary considerably depending on its source and preparation method [6]. Although purified sulforaphane has shown efficacy in preclinical models of neurological disorders, the therapeutic potential of whole-food-based sources, such as broccoli sprout extract (BSE) containing natural sulforaphane precursors, remains poorly characterized [7,8]. Few studies have comprehensively assessed the effects of orally administered broccoli formulations using integrated behavioral, biochemical, and histological endpoints within a single experimental design. This gap highlights the need to evaluate functional food-derived sulforaphanes using models that can better approximate real-world human consumption.

Therefore, this study aimed to evaluate the neuroprotective efficacy of BSE, a dietary source of sulforaphane precursors, using a Scopolamine-induced mouse model of memory impairment. This well-established pharmacological model recapitulates the key pathological features of AD by disrupting cholinergic neurotransmission and is widely used to screen cognitive-enhancing interventions [9]. Special emphasis was placed on its ability to modulate oxidative stress, neuroinflammation, and neurotrophic signaling, which are key mechanisms implicated in Alzheimer’s disease pathology.

## 2. Materials and Methods

### 2.1. Animal Care and Experimental Design

Eight-week-old male C57BL/6J mice were obtained from ORIENT BIO Inc. (Seongnam, Republic of Korea) and acclimated for one week prior to the experiment. The animals were housed in the animal facility of Binaree Inc. (Daegu, Republic of Korea) under specific pathogen-free conditions maintained at 22 ± 3 °C with 30 ± 10% relative humidity, a 12 h light/dark cycle (lights on from 08:00 to 20:00), and illumination of 150–300 lux. The mice were group-housed (≤5 per polycarbonate cage) with free access to standard laboratory chow (Purina Korea, Seoul, Republic of Korea) and filtered tap water. The animals were monitored daily for signs of pain, distress, and abnormal behavior. No mortality or adverse clinical signs were observed during the study, and no humane endpoints were necessary. All animal experiments were performed in accordance with the ethical guidelines and regulations approved by the Institutional Animal Care and Use Committee (IACUC) at Dongguk University, Seoul, Korea (Approval No. IACUC-2023-17).

### 2.2. Preparation and Characterization of BSE

BSE was manufactured and supplied by BK Bio Co., Ltd. (Jeju, Republic of Korea). Briefly, the broccoli sprouts (*Brassica oleracea* var. *italica*, Jeju, Republic of Korea) were dried in a hot-air oven at 60 °C. The dried sprouts were extracted with 20 volumes of 50% ethanol at 55 °C for 4 h under continuous agitation. The resulting extract was filtered and concentrated under reduced pressure at 55 °C. The extract was freeze-dried to obtain a powder, which was stored at −20 °C until use.

The sulforaphane and glucoraphanin contents of BSE were analyzed using high-performance liquid chromatography (HPLC). BSE was prepared at a concentration of 20 mg/mL in 80% ethanol, sonicated in an ultrasonic bath for 30 min, and subsequently filtered through a 0.45 μm PTFE membrane before analysis. Chromatographic separation was performed on a Shim-pack GIS C18 column (4.6 × 250 mm, 5 μm) equipped with a PDA detector set at 190 nm. The mobile phase consisted of acetonitrile and distilled water in gradient mode, with a flow rate of 1.5 mL/min, an injection volume of 10 μL, and a column oven temperature maintained at 40 °C. Glucoraphanin analysis was carried out under similar conditions, except that detection was performed at 225 nm, the mobile phase consisted of acetonitrile and 0.1% phosphoric acid, and the flow rate was adjusted to 0.8 mL/min.

### 2.3. Drug and Sample Administration

Animals randomly assigned to five groups (*n* = 12 per group) using randomized block design: vehicle-treated normal group (Veh group), scopolamine-treated group (SCO group; 1 mg/kg administered intraperitoneally; MilliporeSigma, Burlington, MS, USA), donepezil (8 mg/kg administered orally; MilliporeSigma) + scopolamine group (DON + SCO group), 100 mg/kg BSE (administered orally) + scopolamine group (BR100 + SCO group), and 200 mg/kg BSE (administered orally) + scopolamine group (BR200 + SCO group). Each individual mouse was considered an experimental unit. The sample size was based on previous studies with similar designs. Each group was administered the substance once daily, five times per week for four consecutive weeks via oral gavage using distilled water as the vehicle. BSE was reconstituted in distilled water to prepare suspensions of 15 mg/mL and 30 mg/mL, corresponding to doses of 100 and 200 mg/kg body weight, respectively. The suspensions were administered once daily via oral gavage at a volume of 6.67 mL/kg. The 8 mg/kg oral dose of donepezil was selected based on previous reports demonstrating significant inhibition of acetylcholinesterase (AChE) in the cortex and hippocampus of mice at this concentration [10]. Following this 4-week pretreatment period, all groups, except the normal group, received intraperitoneal injections of scopolamine (1 mg/kg) once daily during the behavioral testing phase. The scopolamine model was chosen because it has been shown to cause memory impairment through cholinergic blockade. Behavioral testing and analyses were performed by a blinded researcher. Confounding factors were not controlled for. No animals or data points were excluded from the experiments. All the animals and measurements were included in the final analyses without omission.

### 2.4. Behavioral Assessments

#### 2.4.1. Passive Avoidance Test

The step-through passive avoidance test was performed using a shuttle box apparatus (Ugo Basile, Gemonio, Italy) controlled by dedicated behavioral software. The apparatus consisted of light and dark compartments separated by a guillotine door. Behavioral data were automatically recorded via the software. During the training session, each mouse was placed in an illuminated compartment. After a 30 s acclimation period, the door separating the two compartments was opened. Upon entry into the dark compartment, the door was closed, and a mild foot shock (0.5 mA, 2 s) was delivered through the grid floor. The cut-off time for the training session was set at 180 s. After shock delivery, the animals were returned to their home cages after a 10 s rest period in a recovery cage. Twenty-four hours after training, the mice underwent a test session. The procedure was identical except for the foot shock intensity, which was reduced to 0.1 mA. The latency time to enter the dark compartment (step-through latency) was recorded, with a cut-off time of 300 s. Longer latency times were interpreted as better memory retention.

#### 2.4.2. Y-Maze Test

Spontaneous spatial alternation behavior was evaluated using a Y-maze apparatus consisting of three arms (42 cm long, 3 cm wide, and 12 cm high) arranged at an angle of 120°. The maze was made of black polyvinyl plastic, and the arms were labeled A, B, and C in a random order. Each mouse was placed at the end of an arm and allowed to explore the maze freely for 8 min. An arm entry was defined as the mouse entering an arm completely with all four limbs and the tail inside the arm. Reentries into the same arm were recorded separately. Alternation behavior was defined as entering all three arms in a different order without repetition. The percentage of spontaneous alternation was calculated as follows:

Alternation (%) = (Number of alternations/[Total arm entries − 2]) × 100.

### 2.5. Blood Collection and Plasma Cytokine Analysis

Mice were anesthetized with sodium pentobarbital, and blood samples were collected from the retro-orbital plexus using EDTA-treated Pasteur pipettes. The collected blood was centrifuged at 2600× *g* for 15 min at 4 °C, and the plasma was separated and stored at −20 °C until analysis. Plasma levels of pro-inflammatory cytokines, including tumor necrosis factor-α (TNF-α) and interleukin-6 (IL-6), were quantified using commercial ELISA kits (Endogen Inc., Cambridge, MA, USA) according to the manufacturer’s instructions.

### 2.6. Brain Tissue Homogenization and Biochemical Assays

The cortical and hippocampal tissues were quickly dissected and rinsed in phosphate-buffered saline (PBS, pH 7.4; Welgene Inc., Gyeongsan, Republic of Korea) to remove any remaining blood. Homogenates were prepared on ice and centrifuged to obtain clear supernatants for subsequent analysis. Homogenates were prepared on ice and centrifuged to obtain clear supernatants, and all procedures were performed according to the manufacturer’s instructions. The concentration of brain-derived neurotrophic factor (BDNF) was quantified using the Mouse BDNF ELISA Kit (A1657, Antibodies.com LLC, St. Louis, MO, USA). AChE activity was determined with the Acetylcholinesterase Assay Kit (ab138871, Abcam Limited, Cambridge, UK). Malondialdehyde (MDA) levels were measured using the Lipid Peroxidation [MDA] Assay Kit (ab118970, Abcam Limited). Glutathione (GSH) levels were assessed using the Glutathione Detection Assay Kit (ab65322, Abcam Limited).

### 2.7. Brain Tissue Processing and Immunohistochemistry

Brain tissue samples were fixed in 4% paraformaldehyde at 4 °C overnight and sectioned into 0.1-mm-thick coronal slices using a vibratome (VT1200, Leica, Wetzlar, Germany). For enhanced antibody penetration, sections were processed using the Binaree Tissue Clearing protocol (HRTI-001, Binaree Inc.), which included sequential incubation in Tissue Clearing Solutions A and B at 37 °C for 24 h in a shaking incubator. Immunostaining was performed using the following primary antibodies: anti-choline acetyltransferase (anti-ChAT, 1:100; Dako Agilent Technologies, Santa Clara, CA, USA), anti-cyclooxygenase-2 (anti-COX-2, 1:100; Abcam Limited), and anti-TNF-α (1:100, Abcam Limited). The sections were incubated with primary antibodies at room temperature overnight, followed by appropriate secondary antibody staining. Finally, the tissues were incubated in mounting and storage solutions (Binaree Inc.) for 24 h to match the refractive index before imaging.

### 2.8. Statistical Analysis

All data are expressed as the mean ± standard deviation (SD). Effect sizes and confidence intervals (CIs) were not calculated. Statistical analyses were performed using GraphPad Prism software (version 10.2.3; GraphPad Software Inc., San Diego, CA, USA). Differences among multiple groups were assessed using a one-way analysis of variance (ANOVA) under the assumption of a Gaussian distribution and equal standard deviations. followed by Tukey’s post hoc test. A *p*-value < 0.05 was considered statistically significant.

## 3. Results

### 3.1. Quantification of Sulforaphane and Glucoraphanin

Quantitative analysis revealed glucoraphanin to be the predominant compound in BSE, accounting for 3.21% (32,067.7 mg/kg) of dry weight. Sulforaphane was detected at 0.29% (2905.3 mg/kg). Both compounds were determined as indicated by low RSD values (<1%).

### 3.2. Effects of BSE on Body Weight and General Health Parameters

**Figure 1:**
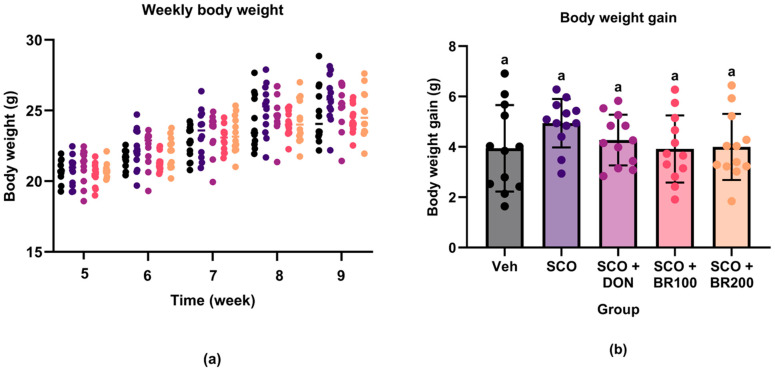
Changes in body weight during the experimental period: (**a**) weekly body weight of mice during the 4-week oral administration; (**b**) body weight gain calculated as the difference between final and initial body weights. Data are presented as the mean ± standard deviation (SD) (*n* = 12). Statistical analysis was performed using one-way ANOVA followed by Tukey’s post hoc test. Different lowercase letters indicate significant differences (*p* < 0.05).

Body weight was monitored throughout the four-week oral administration period to evaluate the effects of the treatments on general health. All groups exhibited a similar pattern of gradual weight gain, with no significant differences between them (Figure 1a). Consistently, total weight gain did not differ significantly among the groups (Figure 1b). These results suggested that none of the treatments induced adverse changes in body weight, indicating no apparent systemic burden under the experimental conditions.

### 3.3. Anti-Inflammatory Effects of BSE at Plasma and Hippocampal Levels

**Figure 2:**
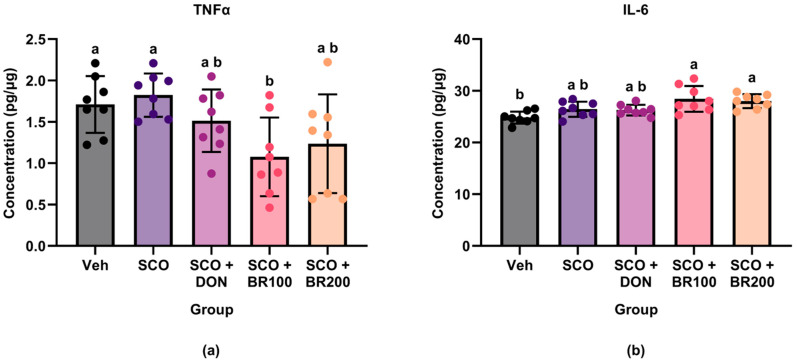
Effects of treatment on plasma levels of pro-inflammatory cytokines: (**a**) TNF-α and (**b**) IL-6 concentrations in mouse plasma (measured by ELISA). Data are presented as the mean ± SD (*n* = 8). Statistical analysis was performed using one-way ANOVA followed by Tukey’s post hoc test. Different lowercase letters indicate significant differences between groups (*p* < 0.05).

**Figure 3:**
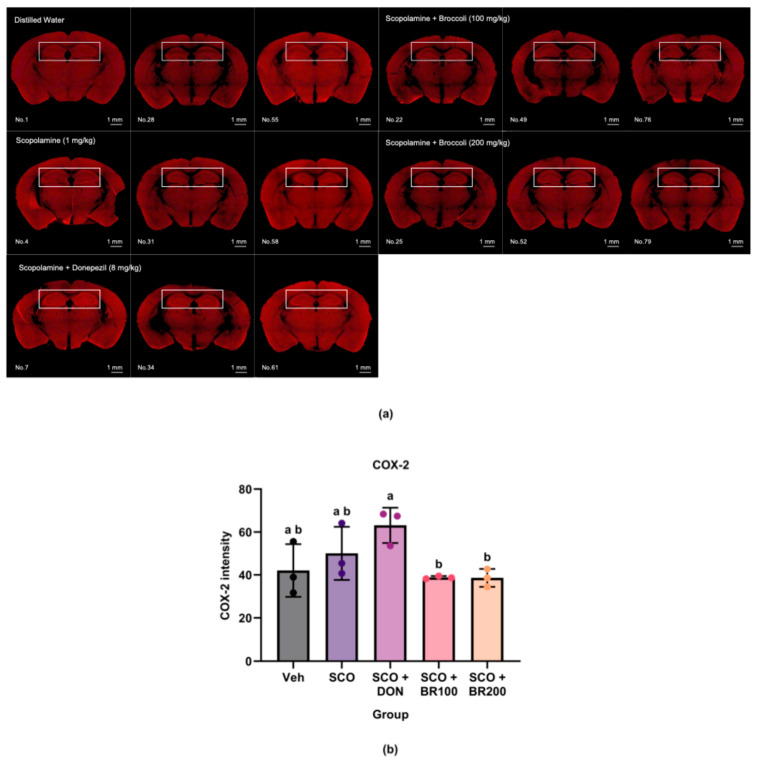
Effects of treatment on cyclooxygenase-2 (COX-2) expression in the hippocampus: (**a**) representative immunohistochemical images of COX-2 expression in the hippocampus (magnification ×4); (**b**) quantification of COX-2-positive signals. Data are presented as mean ± SD (*n* = 3). Statistical significance was assessed using one-way ANOVA followed by Tukey’s post hoc test. Different lowercase letters denote significant differences between groups (*p* < 0.05).

**Figure 4:**
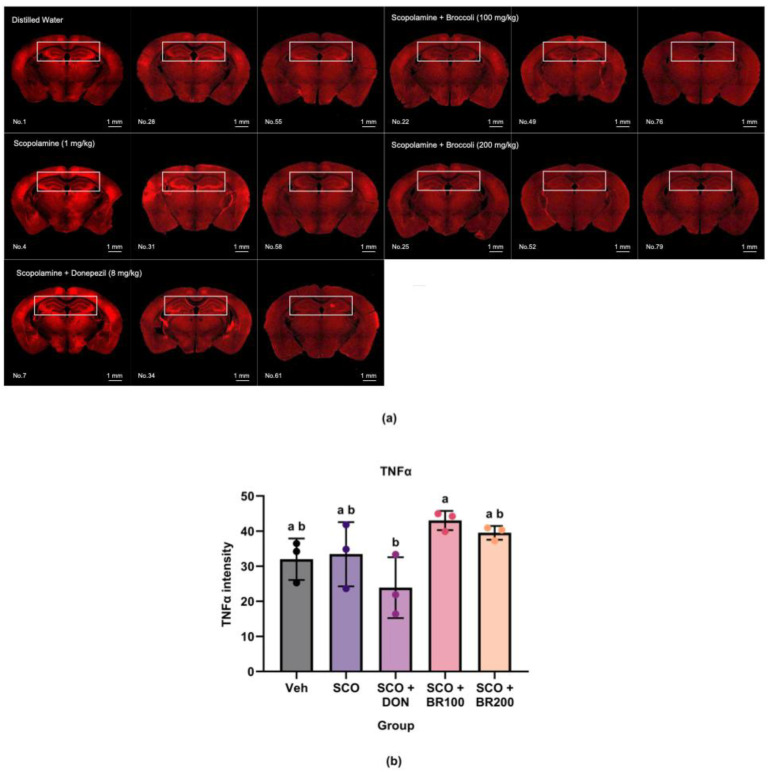
Effects of treatment on tumor necrosis factor-α (TNF-α) expression in the hippocampus: (**a**) representative immunohistochemical images of TNF-α expression in the hippocampus (magnification ×4); (**b**) quantification of TNF-α-positive signals. Data are presented as the mean ± SD (*n* = 8). Statistical significance was assessed using one-way ANOVA followed by Tukey’s post hoc test. Different lowercase letters denote significant differences between groups (*p* < 0.05).

The plasma and hippocampal levels of pro-inflammatory markers were measured to assess the anti-inflammatory effects of BSE. Compared to both the vehicle and scopolamine groups, the BR100 + SCO group significantly decreased plasma TNF-α levels (Figure 2a). However, the IL-6 levels did not differ significantly between the SCO and BSE-treated groups, although the BR100 + SCO and BR200 + SCO groups exhibited higher levels than the vehicle group (Figure 2b). In the hippocampus, cyclooxygenase-2 (COX-2) expression was significantly lower in the BR100 + SCO and BR200 + SCO groups than in the DON + SCO group. No significant differences were observed between the vehicle and scopolamine groups (Figure 3). In contrast, hippocampal TNF-α expression was significantly higher in the BR100 + SCO group than in the DON + SCO group, with no significant change in the BR200 group (Figure 4). These results suggest that the inflammatory responses to BSE may differ depending on the specific marker used.

### 3.4. Modulatory Effects on Neurotrophic and Cholinergic Systems in the Brain

#### 3.4.1. Restoration of BDNF Levels

**Figure 5:**
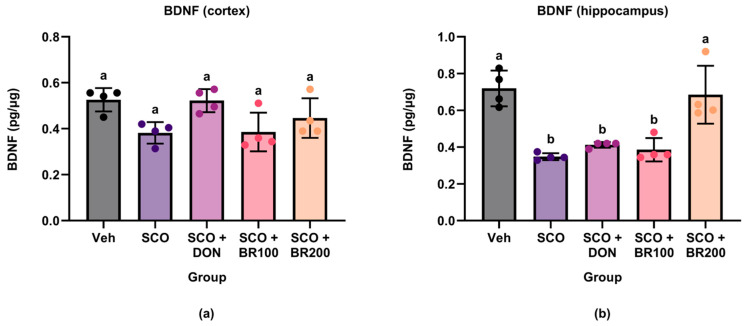
Effects of treatment on brain-derived neurotrophic factor (BDNF) levels in mouse brain regions. BDNF concentrations were measured in the (**a**) cortex and (**b**) hippocampus using ELISA. Data are shown as the mean ± SD (*n* = 8). Statistical analysis was performed using one-way ANOVA followed by Tukey’s post hoc test. Different lowercase letters indicate significant differences between groups (*p* < 0.05).

BDNF levels were quantified in the cortex and hippocampus to investigate the neurotrophic effects of the BSE. No significant differences were observed in cortical BDNF levels among the experimental groups (Figure 5a). In contrast, a significant decrease in hippocampal BDNF levels was observed in the scopolamine group compared to the vehicle group. Notably, this decrease was reversed by treatment with BR200 (Figure 5b). These results suggest that high-dose BSE may attenuate the scopolamine-induced reductions in hippocampal BDNF expression.

#### 3.4.2. Regulation of AChE Activity

**Figure 6:**
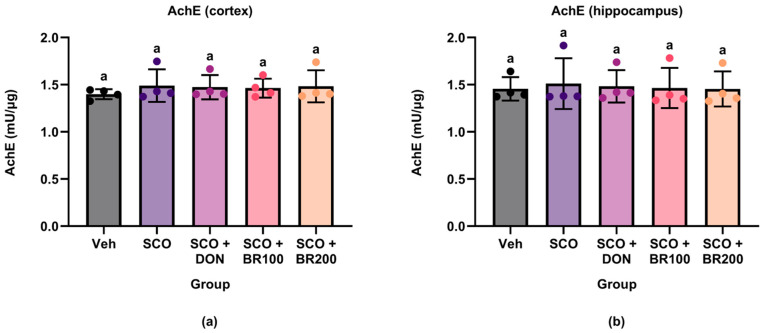
Effects of treatment on acetylcholinesterase (AChE) activity in mouse brain regions. AChE activity was quantified in (**a**) cortex and (**b**) hippocampus using a colorimetric assay. Data are presented as the mean ± SD (*n* = 4). Statistical significance was assessed using one-way ANOVA followed by Tukey’s post hoc test. Different lowercase letters denote significant differences between groups (*p* < 0.05).

To assess the effect of the BSE on cholinergic function, AChE activity was measured in the cortex and hippocampus. No significant differences were observed between groups in either brain region (Figure 6). These findings suggest that BSE did not alter AChE activity under the experimental conditions employed in this study.

#### 3.4.3. Preservation of Choline Acetyltransferase (ChAT) Expression in the Striatum

**Figure 7:**
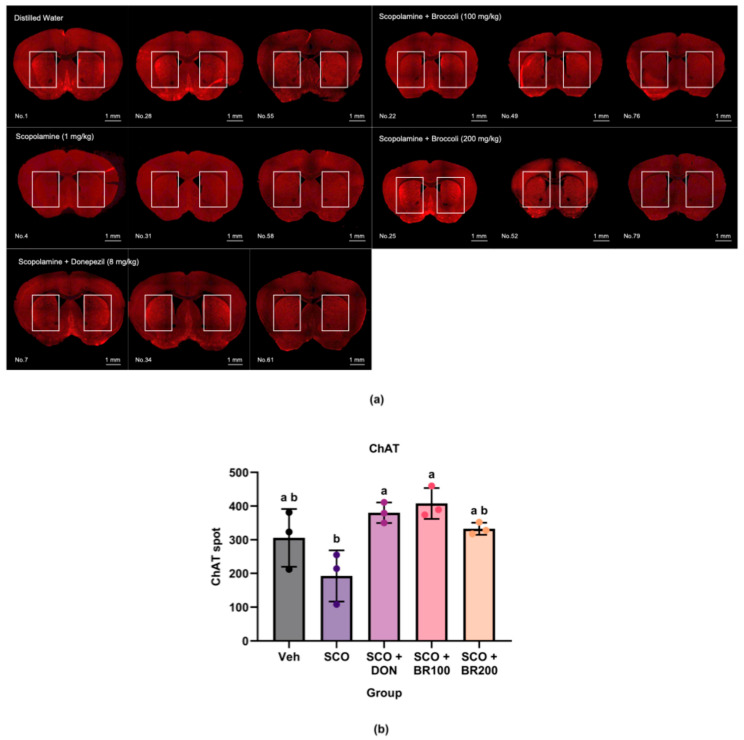
Effects of treatment on choline acetyltransferase (ChAT) expression in the striatum: (**a**) representative immunohistochemical images of ChAT expression in the striatum. The boxed area indicates the striatal region; (**b**) quantification of ChAT-positive cells. Data are presented as the mean ± SD (*n* = 3). Statistical significance was assessed using one-way ANOVA followed by Tukey’s post hoc test. Different lowercase letters denote significant differences between groups (*p* < 0.05).

The neuroprotective effects of the BSE on cholinergic neurons were examined by assessing ChAT expression in the striatum using immunohistochemistry. Scopolamine treatment led to a significant reduction in the number of ChAT-positive cells compared to that in the DON + SCO and BR100 + SCO groups, indicating a cholinergic dysfunction. This reduction was significantly reversed by treatment with donepezil or BSE (100 mg/kg), whereas BSE (200 mg/kg) had no significant effect (Figure 7).

### 3.5. Antioxidant Properties of BSE in Cortical and Hippocampal Regions

#### 3.5.1. Reduction of Lipid Peroxidation as Indicated by MDA Levels

**Figure 8:**
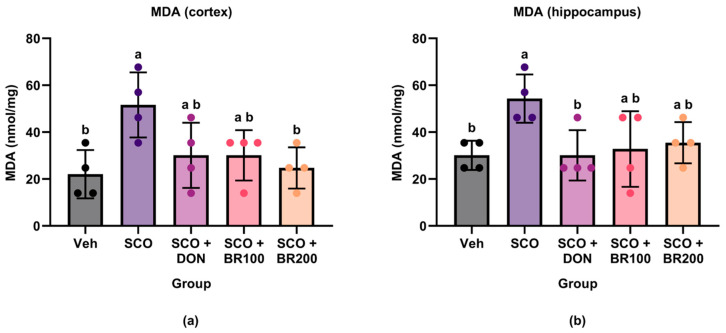
Effects of treatment on malondialdehyde (MDA) levels in mouse brain regions. MDA concentrations were quantified in the (**a**) cortex and (**b**) hippocampus by ELISA to assess lipid peroxidation. Data are presented as the mean ± SD (*n* = 4). Statistical significance was assessed using one-way ANOVA followed by Tukey’s post hoc test. Different lowercase letters denote significant differences between groups (*p* < 0.05).

Lipid peroxidation was evaluated by measuring MDA levels in the cortex and hippocampus. Scopolamine administration significantly increased MDA levels in the cortex compared to the vehicle group. This increase was partially offset by the DON + SCO and the BR100 + SCO group and was substantially reduced by the BR200 + SCO group (Figure 8a). Similarly, the MDA levels were significantly elevated in the hippocampus following scopolamine treatment. However, a significant reduction was only observed in the DON + SCO group. Although the BSE treatment showed a decreasing trend, this did not reach statistical significance (Figure 8b).

#### 3.5.2. Assessment of GSH Levels

**Figure 9:**
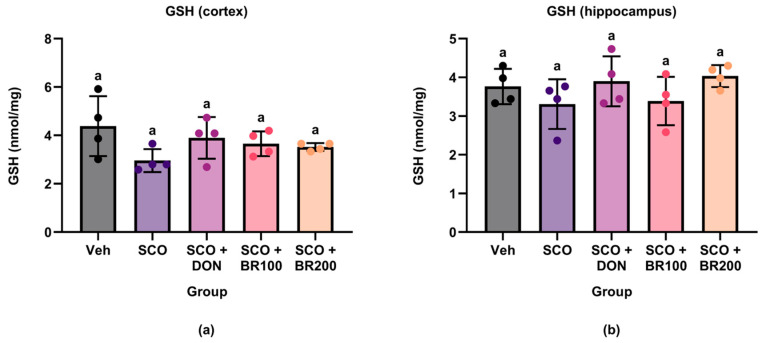
Effects of treatment on glutathione (GSH) levels in mouse brain regions. GSH concentrations were quantified in the (**a**) cortex and (**b**) hippocampus by ELISA to evaluate antioxidant capacity. Data are presented as the mean ± SD (*n* = 4). Statistical significance was assessed using one-way ANOVA followed by Tukey’s post hoc test. Different lowercase letters denote significant differences between groups (*p* < 0.05).

To evaluate the antioxidant effects of the BSE, GSH levels were measured in the cortex and hippocampus. No significant differences in the GSH concentrations were observed between the experimental groups in either brain region (Figure 9). These results suggest that the BSE did not affect endogenous GSH levels under these experimental conditions.

### 3.6. Cognitive-Enhancing Effects of BSE in Behavioral Tests

**Figure 10:**
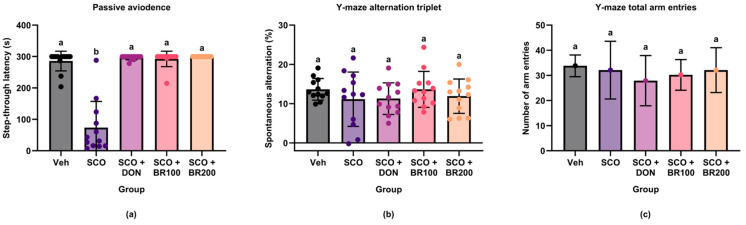
Effects of treatment on cognitive performance in scopolamine-induced memory-impaired mice: (**a**) step-through latency in the passive avoidance test; (**b**) spontaneous alternation (%) in the Y-maze test; (**c**) total number of arm entries in the Y-maze test. Data are presented as the mean ± SD (*n* = 12). Statistical significance was assessed using one-way ANOVA followed by Tukey’s post hoc test. Different lowercase letters denote significant differences between groups (*p* < 0.05).

Passive avoidance and Y-maze tests were conducted to evaluate the cognitive effects of the BSE. Scopolamine treatment significantly decreased step-through latency in the passive avoidance test, indicating impaired long-term memory, compared to the vehicle group. Donepezil and BSE treatments significantly improved this deficit, restoring the latency to levels comparable to those in the vehicle group (Figure 10a).

In the Y-maze test, no significant differences in spontaneous alternation percentages were observed between the groups (Figure 10b), suggesting that spatial working memory remained unaffected by the current conditions. Similarly, the total number of arm entries did not differ significantly (Figure 10c), indicating that the locomotor activity was unaffected by scopolamine or the test substances.

## 4. Discussion

Body weight monitoring is a common parameter used to evaluate the general health and systemic toxicity in animal studies [11]. In the present study, repeated oral administration of BSE over four weeks was well tolerated, as evidenced by the absence of significant changes in body weight or adverse physiological effects. These results are consistent with those of previous studies reporting the safety of broccoli and its bioactive compounds in vivo. For instance, no abnormal changes in body weight, food intake, or organ indices were observed in rodents administered broccoli seeds [12].

BSE was administered at doses of 100 and 200 mg/kg. The sulforaphane precursor content in mature broccoli typically ranges from 44 to 171 mg per 100 g dry weight [13]. These doses are markedly lower than the reported LD_50_ (~212.67 mg/kg) and TD_50_ (~191.58 mg/kg) of purified sulforaphane in mice, suggesting a safe dosage range [14]. The absence of weight loss and signs of toxicity suggests that BSE does not impair gastrointestinal function or appetite. Stable body weight across groups minimizes the potential confounding factors related to animal stress or health deterioration, thereby supporting the reliability of subsequent behavioral and biochemical outcomes.

Previous studies have reported that scopolamine induces neuroinflammation by increasing the production of pro-inflammatory mediators in the hippocampus [15,16]. However, in the present study, scopolamine administration did not consistently elevate systemic or hippocampal inflammatory markers. Serum TNF-α, hippocampal COX-2, and hippocampal TNF-α levels remained unchanged, and only a modest increase in Serum IL-6 was observed. Notably, IL-6 levels were significantly higher in the BR100 + SCO group and the BR200 + SCO group than in the vehicle group, though not significantly different from the SCO group. These results suggest that scopolamine did not markedly elevate IL-6 levels and that BSE treatment did not exacerbate IL-6 expression beyond the level induced by scopolamine. These results suggest that the inflammatory response to scopolamine depends on the dose and duration of exposure and that BSE has cytokine- and tissue-specific regulatory effects.

Donepezil, a cholinesterase inhibitor, is known for its anti-inflammatory effects through the cholinergic anti-inflammatory pathway [17]. This study found that donepezil significantly reduced TNF-α levels in the hippocampus. However, it failed to suppress serum TNF-α and IL-6 levels and unexpectedly increased COX-2 expression in the hippocampus. These results suggested that the anti-inflammatory effects of donepezil were not uniformly exerted across inflammatory mediators or tissues. The limited effects of donepezil observed in this study may be due to several factors. While an oral dose of 8 mg/kg has been shown to inhibit acetylcholinesterase activity in the cortex and hippocampus of mice, the efficacy of this dose depends on the duration of treatment and individual biomarker sensitivity. The four-week administration regimen used in this study may not have been sufficient to produce consistent effects in the systemic and central compartments. Additionally, the scopolamine-induced model primarily reflects acute cholinergic blockade rather than chronic neurodegeneration [9]. This may have prevented the full manifestation of donepezil’s pharmacological properties.

Even in the absence of strong inflammatory stimuli, the BSE exhibited selective immunomodulatory activity. The BR100 + SCO group significantly decreased serum TNF-α levels, and the BR100 + SCO and the BR200 + SCO groups suppressed hippocampal COX-2 expression more effectively than donepezil did. These results align with the cytokine-specific regulatory actions of sulforaphane-rich compounds, which modulate inflammation by activating NRF2 and inhibiting NF-κB signaling [8,18]. However, serum IL-6 levels remained unchanged, and TNF-α expression in the hippocampus increased in the BR100 group. This discrepancy between systemic and hippocampal responses may reflect differential regulation of peripheral versus central inflammatory pathways. Plasma cytokines are primarily influenced by systemic immune cells, whereas hippocampal TNF-α expression is largely shaped by local microglial activation and blood-brain barrier dynamics [19].

High-dose broccoli significantly restored hippocampal BDNF levels, suggesting the involvement of sulforaphane-mediated neurotrophic pathways mediated by sulforaphane. Sulforaphane activates NRF2 and CREB signaling, leading to increased BDNF transcription and neuroprotection under stressful conditions [20]. These mechanisms are particularly relevant in neurodegenerative diseases, in which BDNF depletion contributes to synaptic dysfunction and cognitive decline. Thus, the observed effects may reflect the disease-modifying potential of the BSE by enhancing endogenous neurotrophins.

Despite the observed cognitive improvement, AChE activity remained unaltered in both the broccoli- and donepezil-treated groups. This suggests that neither scopolamine nor the treatments significantly affected the cholinesterase activity under the current experimental conditions. While some studies have reported that sulforaphane reduces AChE activity and increases acetylcholine levels [21], others have reported no changes in AChE or ChAT activity despite observing functional benefits [22]. These discrepancies likely reflect the differences in dosage, treatment duration, or region-specific responses.

Interestingly, a low dose of broccoli extract (BR100) preserved striatal ChAT expression, whereas a high dose was ineffective. ChAT is a rate-limiting enzyme in the biosynthesis of acetylcholine and a key marker of cholinergic neuronal integrity. This loss is closely associated with cognitive impairments in neurodegenerative diseases [22]. Collectively, these findings suggest that sulforaphane-rich BSE exerts cognitive benefits through a multifaceted mechanism involving the enhancement of hippocampal BDNF expression and partial preservation of cholinergic neuronal markers. This multi-targeted profile highlights its potential as a dietary neuroprotective agent, particularly in early-stage interventions for cognitive decline.

BSE exhibited region-specific antioxidant effects in a scopolamine-induced model. Significant reductions in cortical MDA levels were observed following the high-dose treatment (200 mg/kg), although no significant changes were observed in the hippocampus. This differential response may reflect variations in the regional oxidative vulnerability, baseline antioxidant capacity, and the bioavailability of broccoli-derived compounds. Sulforaphane, a key isothiocyanate found in broccoli, activates the NRF2 signaling pathway and induces antioxidant enzymes such as HO-1 and NQO1 [23,24]. Although NRF2 activation was not directly assessed in the present study, the observed cortical protection may involve these enzymatic defense mechanisms.

In contrast, GSH levels remained unchanged in both brain regions, suggesting that the antioxidant effect was not mediated by the direct modulation of the GSH pool. Previous clinical and preclinical studies have demonstrated that sulforaphane can increase systemic and brain GSH levels [25]. However, owing to the rapid turnover and strict regulation of GSH homeostasis, it is reasonable to speculate that the observed protection occurs primarily through inducible enzyme systems [26]. Taken together, these findings suggest that BSE provides partial cortical antioxidant protection via non-GSH-dependent mechanisms involving sulforaphane-induced enzymatic pathways.

This study demonstrated that BSE significantly improved scopolamine-induced cognitive deficits in mice. Specifically, the extract enhanced long-term memory performance in a passive avoidance task. This effect is comparable to that observed with donepezil, a well-known cholinesterase inhibitor. These results suggest that BSE exhibits potent neuroprotective properties in a cholinergic dysfunction model. These results align with prior research indicating that BSEs rich in sulforaphane can improve memory performance in similar models [27,28].

Interestingly, BSE neither improved spatial working memory performance in the Y-maze test nor affected locomotor activity. However, the selective enhancement of passive avoidance performance suggests that broccoli-derived compounds may target hippocampus-dependent memory processes more effectively. Previous studies have shown that passive avoidance depends heavily on hippocampal integrity, whereas Y-maze alternations are more closely associated with prefrontal cortical function [29,30]. The absence of hyperlocomotion indicates that the observed behavioral effects were not due to nonspecific increases in arousal or motor activity [27].

Collectively, these findings highlight the potential of BSE as a dietary intervention for memory impairment, particularly in the hippocampal memory systems. The region-specific effects observed, as well as the mechanistic alignment with antioxidant and anti-inflammatory pathways, underscore the therapeutic potential of sulforaphane-rich plant extracts. Although this hypothesis requires validation through clinical trials, preliminary evidence from rodent studies suggests that these extracts can benefit human cognitive health. Nevertheless, this study has certain limitations that should be considered. Although the scopolamine model is commonly used to mimic the cholinergic deficits in AD, it does not completely replicate the intricate pathophysiology of chronic neurodegeneration. Key molecular pathways, such as NRF2 activation, were not evaluated directly. Additionally, quantitative validation of memory-related gene expression and signaling pathways, as well as comprehensive behavioral analyses, such as the Morris water maze, were not performed. Future studies should address these limitations to provide a more comprehensive understanding of the molecular mechanisms and behavioral outcomes associated with BSE.

## 5. Conclusions

This study demonstrates that oral administration of BSE provides neuroprotective effects in a scopolamine-induced memory impairment model. These effects are achieved by alleviating oxidative stress and supporting neurotrophic signaling. BSE reduced systemic and hippocampal inflammation, as evidenced by lower TNF-α and COX-2 levels. However, these effects depended on the marker and the dose used. No significant changes were observed in the AChE activity, GSH levels, or spatial working memory performance in the Y-maze test. Nevertheless, passive avoidance testing revealed that BSE improved the scopolamine-induced long-term memory deficits. Taken together, these results suggest that BSE may improve cognition by modulating oxidative and inflammatory responses as well as the neurotrophic and cholinergic systems. Further studies are required to identify the active constituents responsible for these effects and validate their efficacy in chronic or age-related neurodegenerative models.

## Data Availability

The original contributions presented in the study are included in the article, further inquiries can be directed to the corresponding author.

## Abbreviations

The following abbreviations are used in this manuscript:

AChE	Acetylcholinesterase
AD	Alzheimer’s disease
ANOVA	Analysis of variance
BDNF	Brain-derived neurotrophic factor
BSE	Broccoli sprout extract
ChAT	Choline acetyltransferase
COX-2	Cyclooxygenase-2
EDTA	Ethylenediaminetetraacetic acid
GSH	Glutathione
IACUC	Institutional Animal Care and Use Committee
IL-6	Interleukin-6
MDA	Malondialdehyde
NRF2	Nuclear factor erythroid 2
TNF-α	Tumor necrosis factor-alpha
